# A cross-sectional study of well water arsenic and child IQ in Maine schoolchildren

**DOI:** 10.1186/1476-069X-13-23

**Published:** 2014-04-01

**Authors:** Gail A Wasserman, Xinhua Liu, Nancy J LoIacono, Jennie Kline, Pam Factor-Litvak, Alexander van Geen, Jacob L Mey, Diane Levy, Richard Abramson, Amy Schwartz, Joseph H Graziano

**Affiliations:** 1 Department of Psychiatry, College of Physicians and Surgeons, Columbia University New York, NY, USA; 2 New York State Psychiatric Institute New York, NY, USA; 3 Departments of Environmental Health Sciences, Epidemiology and Biostatistics, Columbia University Mailman School of Public Health New York, NY, USA; 4 Lamont-Doherty Earth Observatory of Columbia University Palisades, NY, USA; 5 City University of New York, Kingsborough CC Brooklyn, NY, USA; 6 Formerly with Readfield, ME Public Schools Readfield, USA; 7 University of New Hampshire, NH Institute for Health Policy & Practice Durham, NH, USA; 8 Division of Child and Adolescent Psychiatry, NYSPI, 1051 Riverside Drive, New York, NY 10032, USA

**Keywords:** Arsenic, Child intelligence, Well water, WISC-IV, Working Memory

## Abstract

**Background:**

In recent studies in Bangladesh and elsewhere, exposure to arsenic (As) via drinking water is negatively associated with performance-related aspects of child intelligence (e.g., Perceptual Reasoning, Working Memory) after adjustment for social factors. Because findings are not easily generalizable to the US, we examine this relation in a US population.

**Methods:**

In 272 children in grades 3–5 from three Maine school districts, we examine associations between drinking water As (WAs) and intelligence (WISC-IV).

**Results:**

On average, children had resided in their current home for 7.3 years (approximately 75% of their lives). In unadjusted analyses, household well WAs is associated with decreased scores on most WISC-IV Indices. With adjustment for maternal IQ and education, HOME environment, school district and number of siblings, WAs remains significantly negatively associated with Full Scale IQ and Perceptual Reasoning, Working Memory and Verbal Comprehension scores. Compared to those with WAs < 5 μg/L, exposure to WAs ≥ 5 μg/L was associated with reductions of approximately 5–6 points in both Full Scale IQ (p < 0.01) and most Index scores (Perceptual Reasoning, Working Memory, Verbal Comprehension, all *p’s* < 0.05). Both maternal IQ and education were associated with lower levels of WAs, possibly reflecting behaviors (e.g., water filters, residential choice) limiting exposure. Both WAs and maternal measures were associated with school district.

**Conclusions:**

The magnitude of the association between WAs and child IQ raises the possibility that levels of WAs ≥ 5 μg/L, levels that are not uncommon in the United States, pose a threat to child development.

## Background

Previously, we described dose-dependent adverse associations between consumption of arsenic (As)- contaminated water from household wells and intellectual function in young Bangladeshi children [1-3]. In our initial work with 6- [1] and 10-year olds [2], after adjustment for social factors related to intellectual function, water arsenic concentration (WAs) was significantly negatively related to WPPSI-III and WISC-III Performance (nonverbal ability) scores, but, in most instances, not to other components of intelligence, such as Verbal scores. In a more recent study of Bangladeshi 9–10 year olds [3], associations between WAs and both Verbal Comprehension and Working Memory scores from the WISC-IV remained marginally significant after adjustment for socio-demographic features and for co-occurring exposure to manganese in drinking water; WAs was unrelated to other aspects of intellectual functioning. In a neighboring region in West Bengal [4], and in two studies of children residing near metallurgic complexes in Mexico [5,6], urinary arsenic (UAs) concentration was negatively related to some subtests in earlier WISC versions. In a small study relating hair levels of various metals to US children’s intelligence [7], As concentration in hair was associated with poorer verbal learning and memory scores. In a recent large study in Bangladesh [8], at age 5 years, both Full Scale IQ (FSIQ) and Verbal intelligence assessed on the WPPSI-III [9] were negatively associated with WAs exposure. In sum, while several studies in different populations suggest that As exposure may affect early development, there is little consistency in the specific components of child intelligence most affected.

Generalizing these findings to US children is problematic. First, assessments of children’s intelligence in Bangladesh and West Bengal (and elsewhere where As exposure is high) rely on tests that have been standardized to US populations, but not to local children. Thus, researchers have been unable to estimate deficits in terms of “IQ points lost”. Moreover, in our previous work in Bangladesh, IQ tests were slightly modified to increase cultural appropriateness, so that they are not precisely the same test administered in Western settings. In many such regions, children’s nutritional and health status, as well as their regularity of school attendance, differs greatly from that for US children. Finally, WAs concentrations in previously studied settings are far higher than in US communities, limiting extrapolations to the lower concentrations found in the US. Accordingly, to examine the generalizability of findings from other global settings, we set out to examine the association between As and intelligence in US schoolchildren.

## Methods

### Recruitment and sample

We initially planned to recruit children in grades 3–5 in school districts where prior investigation suggested that the As content of the water supply for some households would be elevated above the EPA Guideline of 10 μg/L [10], and where families used household wells. Recruitment began in 2006–2007, in two New Hampshire (NH) school districts. Examination of well water characteristics for the first 53 recruited NH children revealed very low levels of WAs [on average 2.76 μg/L, with only 5.7% (3 wells) exceeding the US standard of 10 μg/L]. For this reason, in 03/2008, we shifted focus to school districts surrounding Augusta Maine (ME), where colleagues had identified higher exposure [11,12] . This report considers children attending 11 participating elementary schools in three Regional School Units (RSUs) in ME (Districts A, B, and C).

Recruitment was open to those in grades 3–5 in participating schools. Parents received information distributed by the school, informing them of the study and seeking participation, with notices and response cards sent home in children’s backpacks. Families returning a card indicating interest were given more information by study staff by telephone, and then an appointment was scheduled. We excluded children with conditions with known adverse impact on intellectual functioning (e.g., multiple births, neurodevelopmental disorders) and children who were receiving special education services. These restrictions excluded two children with neurodevelopmental conditions (one a “*processing deficit*”, and the other *“delayed cerebral development impacting motor skills and speech”*, both receiving support services at school*)* that would have made our standard assessment difficult to conduct. Parent report of these conditions was confirmed with school personnel. Exclusion likely biased our findings toward the null. We further limited enrollment to those residing at the present address for three or more years.

From the 1595 children who attended grades 3–5 during the assessment period, 581 families (36%) agreed to participate. One child from each family, selected at random, was eligible for inclusion in analyses. Of the 581, appointments were scheduled with the families of 377 children; appointments were not scheduled with the remainder because of scheduling conflicts, inability to contact the family or change in the family’s interest. Of the children with assessments scheduled, 36 were excluded because they were additional children in the same home; two because they were receiving special education services, and 67 because they had not resided in the home for at least three years. We selected this cutoff to balance minimizing the imprecision of defining exposure using home water As level while maximizing sample size, and because few children were expected to have resided in this home since infancy; indeed, only 61 eligible children had resided in this home since their first year of life. This resulted in a final sample of 272 children in three ME school districts and their families, residing in the present home for an average 7.3 years, which represents more than three-fourths of the children’s lifetimes.

### Procedures

Procedures were approved by Columbia University Medical Center and University of New Hampshire Institutional Review Boards. Families received reports summarizing results of well water and developmental testing, information on mitigation procedures (if appropriate) and $25; children received t-shirts.

#### Home interviews

In home visits, we obtained informed parental consent and child assent, collected water samples and children’s toenail samples, and assessed potential covariates, including mothers’ intelligence, home rearing quality, and socioeconomic status.

#### Assessment of child intelligence

In the weeks after the home visit, child intelligence was assessed with the WISC-IV in a quiet school setting. Testers were college graduates, experienced in working with children (two social workers and a former teacher), and trained and supervised by the first author (GW).

#### Water assessments

Water samples were taken at the point of entry into the home (via the connection to the garden hose) and at the consumption point (kitchen sink). For homes using water filters, we took the sample at the post-filtration source. For the point of entry to the home, the home visitor ran the water for 15 minutes, rinsed the collection bottle three times, obtained a 50 ml sample in a polypropylene bottle and sealed the cap. For the point of consumption, the visitor asked how long the family generally let the water run before use, then turned on the faucet and let the water run for that time period, then rinsed and obtained a sample in the same way as for the garden hose. The visitor also inquired about drinking habits, length of residence in the home, well construction and use of filtering procedures.

Laboratory analysis procedures are detailed elsewhere [13,14]. Water samples were stored at room temperature until shipment to Columbia University for analysis. Following 1% per volume acidification in the laboratory with high-purity hydrochloric acid for at least 48 hours [15], samples were analyzed by high-resolution inductively-coupled plasma mass spectrometry (HR-ICP-MS). The analytical detection limit of the method for As is 0.1 μg/L; the standard deviation of a single measurement is estimated at 4 μg/L for concentrations ≤150 μg/L [14]. Coefficients of Variation (CV’s) of As consistency standards (NIST 1640a and NIST 1643e) for daily runs of HR-ICP-MS data in this study are less than 6% (typically 3-4%) and less than 5% over one year for As concentrations of 10 μg/L and higher.

#### Toenail collection and assays

Toenail samples from both feet were collected during home visits, cut with standard nail clippers and placed into envelopes, labeled for right and left feet. Nail collection, washing and digestion [16,17] relied on thorough washing, overnight drying, weighing and digesting in concentrated Ultrex nitric acid. The digested nail samples, diluted to final acid volume of 10%, were analyzed for As using a Perkin-Elmer Elan Dynamic Reaction Cell ICP-MS equipped with a Model AS 93+ autosampler. An ICP-MS- Dynamic Reaction Cell method for metals in nails was developed from a published method [18], with modifications based on suggestions from the Perkin Elmer application laboratory. After calibrating the instrument, and after each batch of 20 samples, we ran quality control nail samples with known As concentrations. During the period when all study samples were analyzed, the intra-precision coefficient of variation for nail As (NAs) in these Quality Control (QC) samples was 2.4%. The inter-precision coefficient of variation for the same QC samples for As was 5.3%. The correlation between NAs concentrations measured from nails on left and right feet was significant [r_(247)_ = 0.86; *p* < 0.001]. NAs concentrations were subsequently averaged. NAs was available for 248 of 272 participants.

### Measures

#### Child intelligence

The Wechsler Intelligence Scale for Children-Fourth Edition (WISC-IV: [19]) is an individually administered assessment of intellectual function, for children 6–16 years old. This revised version of the WISC-III [20] has excellent psychometrics, and provides measures of general intellectual ability (FSIQ) and specific cognitive domains (Verbal Comprehension, Perceptual Reasoning, Working Memory, and Processing Speed Indices).

*Maternal intelligence* was measured on the Wechsler Abbreviated Scale of Intelligence (WASI: [21]), a short and reliable measure of intelligence. It consists of two Performance subtests (Block Design and Matrix Reasoning) and two Verbal (Vocabulary and Similarities) subtests. We examined Vocabulary and Matrix Reasoning subtests.

*Additional sociodemographic characteristics* were assessed during structured interviews with a parent during home visits. We inquired about the number of children (< 18 years old) residing at home, and maternal and paternal age, ethnicity, and education. Reflecting the well-resourced population studied here, predictive analyses stratified maternal and paternal education into two groups, those with and without post-high school education.

*Childrearing characteristics of the home environment* were measured by the HOME Inventory [22], a widely used semi-structured assessment that combines interview and direct observation. The HOME consists of eight factor-analytically derived subscales with acceptable psychometrics that assess the childrearing qualities of the home, tapping constructs, on the Middle Childhood version, such as *Encouragement of Maturity* and *Enrichment*. Low scores reflect less support for child development. Scores have consistently been linked to child intelligence and achievement [22-24]. The HOME Inventory is intended to be administered at home, with both parent and child available. While all families were seen at home, scheduling interviews when both parent and child were available was a challenge, because of the competing demands of after-school programs, parental employment, and because of distances between homes in this rural setting. When children were not present, interviewers did not assess four observational items on the HOME, of 59 possible items. HOME interviews for which 10% or more items (i.e., 6 or more) were not completed were classified as “missing,” allowing their inclusion in our analyses. For participants with fewer than 6 missing HOME items, we extrapolated HOME scores at the subscale level, and used these values to generate prorated total HOME scores. The median for the distribution of HOME scores for 243 individuals missing fewer than 6 items was 53; those with scores ≥ 53 were designated “High” and those < 53 were designated as “Low” scores.

#### Exposure mitigation behaviors

During home visits, mothers were interviewed regarding whether or not their household well had been tested for WAs , whether they used a water filtration system, and whether or not they used bottled water as an alternative source.

Our questions about drinking habits were not detailed enough to determine the amount of tap water consumed.

### Data analysis

We used linear regression analysis to estimate associations between WAs exposure (from the kitchen tap) and child IQ, with and without adjustment for sociodemographic characteristics (i.e., maternal education and intelligence, number of children in the household, qualities of the HOME environment, school district). Preliminary analyses examined bivariate associations between WAs and covariates, including maternal education, maternal intelligence, HOME scores, well characteristics, arsenic mitigation behavior and school district.

Our analysis plan first considered covariate-unadjusted associations between exposure and child intelligence (Model 1). Next, we determined a set of features to be examined in a covariate-adjusted model (Model 2). From the pool of potential covariates, we considered the contributions of those investigated in our prior work on child intelligence and lead [25,26] and arsenic [1,2] exposure, including maternal education and intelligence, number of children in the household, and qualities of the HOME environment. Although maternal education and intelligence were expectably correlated [Spearman r_(270)_ = 0.53, p < 0.0001], both were included in Model 2. Because, as we discuss below, school district was related both to exposure and to other contributors to child intelligence, it was considered in our adjusted models, as well.

In order to describe non-linear patterns, we examined associations between child IQ, with WAs presented both as a continuous (log-transformed) measure, as well as stratified into four categories [WAs < 5 μg/L (n = 141); ≥5 μg/L to <10 (n = 46); ≥10 μg/L to <20 (n = 52); and ≥20 μg/L (n = 33)].

## Results

Table 1 presents socio-demographic and water characteristics for the sample, as well as WISC-IV test results. On average, children were 9.67 years old; had resided in their current home for 7.34 years (i.e., most of their lives); were in 4th grade and there were approximately equal numbers of boys and girls. Overall, participants’ families were in mid-range regarding socio-economic features, with most parents reporting some years of college education. Consistent with the surrounding communities, almost all children were white, and fathers and mothers were, on average, approximately 43 and 41 years old, respectively. On average, WAs measured at the kitchen tap was 9.88 μg/L, with almost a third of samples exceeding the EPA Maximum Contaminant Level of 10 μg/L [27]. The highest level of WAs was 115.3 μg/L. Both maternal and child intelligence were higher than in the overall US general population.

**Table 1:** Sample characteristics

**Measures**	**Mean ± SD or %(n)**
Child characteristics	
Male %	53.31 (145)
Child age	9.67 ± 1.18
Length of current residence	7.34 ± 2.37
School Grade	3.80 ± 0.81
School District	
RSU-A	28.68% (78)
RSU-B	18.75% (51)
RSU-C	52.57% (143)
Race % White	95.2 (259)
Family characteristics	
Paternal age (N = 267)	43.64 ± 6.66
Paternal education	
≤High school graduate	29.04% (79)
Some college	26.84% (73)
College graduate	22.43% (61)
Graduate	19.85% (54)
Any post-graduate	1.84% (5)
Maternal age	40.72 ± 6.02
Maternal education	
≤High school graduate	13.60% (37)
Some college	34.93% (95)
College graduate	32.35% (88)
Any post-graduate	19.12% (52)
Maternal IQ (WASI)	114.84 ± 11.31
HOME Total (n = 243)	52.57 ± 3.73
HOME category	
Missing	10.66 ± 29
Low scores (<53)	40.81 ± 111
High scores (≥53)	48.53 ± 132
Number of other children in home	1.29 ± 0.91
Exposure characteristics	
Overall well water As (μg/L)	9.88 ± 15.06
Water As < 5 μg/L (n = 141)	1.24 ± 1.37
10 > WAs >5 μg/L (n = 46)	7.37 ± 1.34
20 > WAs > 10 μg/L (n = 52)	14.80 ± 3.06
WAs > 20 μg/L (n = 33)	42.55 ± 20.43
% Well water As ≥ 10 μg/L	31.25 (85)
Nail As (μg/g)	4.65 ± 4.60
Child Intelligence	
Full Scale	110.32 ± 12.60
Verbal Comprehension	114.26 ± 15.78
Perceptual Reasoning	108.57 ± 13.25
Working Memory	102.72 ± 12.20
Processing Speed	103.34 ± 13.44

### Covariate-unadjusted associations between well water As and child IQ (Model 1)

Table 2 presents results of regression analyses relating WAs and child intelligence. Before consideration of other covariates, WAs, defined categorically, explained roughly 3-5% of the variance in intelligence test scores. Compared to children with WAs < 5 μg/L, children consuming water ≥ 5 μg/L showed significant reductions in FSIQ, and in all Index scores (Working Memory, Perceptual Reasoning, and Verbal Comprehension) except for Processing Speed. For groups with exposures ≥ 5 μg/L, confidence intervals for IQ points lost overlapped; there are no significant differences among the point estimates for the three exposed groups. When WAs was defined as a continuous measure, its relationship to decrements in IQ scores persisted (data available upon request).

**Table 2:** Predicting child IQ from Water As measured from the kitchen tap

**Contributors**	**Full scale B ± se**	**Working memory B ± se**	**Perceptual reasoning B ± se**	**Verbal comprehension B ± se**	**Processing speed B ± se**
**Model 1: Without adjustment**					
Water As < 5 μg/L					
10 > WAs ≥5 μg/L	−6.96 ± 2.10^**^	−5.24 ± 2.26*	−5.94 ± 2.21^**^	−7.54 ± 2.65^**^	−1.34 ± 2.08
20 > WAs ≥ 10 μg/L	−4.57 ± 2.00^*^	−2.54 ± 2.16	−6.68 ± 2.11^**^	−3.40 ± 2.53	−0.61 ± 1.99
WAs ≥ 20 μg/L	−4.63 ± 2.39^#^	−6.47 ± 2.57*	−3.97 ± 2.51	−3.49 ± 3.02	0.06 ± 2.37
R^2^	5.03%	3.50%	5.02%	3.14%	0.18%
**Model 2: Adjusting for other features**					
Water As < 5 μg/L					
10 > WAs ≥5 μg/L	−6.09 ± 1.98**	−4.88 ± 2.24*	−4.97 ± 2.14*	−6.22 ± 2.49*	−1.74 ± 2.09
20 > WAs ≥ 10 μg/L	−3.15 ± 1.91	−1.13 ± 2.16	−5.10 ± 2.06*	−1.86 ± 2.39	−1.15 ± 2.01
WAs ≥ 20 μg/L	−2.51 ± 2.29	−5.07 ± 2.59^#^	−2.29 ± 2.47	−0.82 ± 2.88	0.40 ± 2.42
Number of other children in home	−0.17 ± 0.79	0.84 ± 0.89	0.36 ± 0.85	−0.72 ± 0.99	−0.84 ± 0.83
Maternal IQ	0.27 ± 0.07***	0.23 ± 0.08**	0.25 ± 0.08***	0.32 ± 0.09***	−0.04 ± 0.07
Maternal Ed (> HS)	4.35 ± 1.62**	0.32 ± 1.84	1.42 ± 1.75	7.62 ± 2.04***	3.14 ± 1.72^#^
HOME					
Missing 6+ items	−5.16 ± 2.54*	−3.34 ± 2.87	−7.44 ± 2.74**	−3.96 ± 3.19	−0.70 ± 2.68
Low scores	−1.96 ± 1.58	−2.21 ± 1.79	−2.45 ± 1.71	0.22 ± 1.99	−1.95 ± 1.67
School district (compared to “C”)					
District “A”	−1.70 ± 1.71	0.47 ± 1.94	0.22 ± 1.84	−3.26 + 2.15	−3.71 + 1.80*
District “B”	1.20 ± 1.97	−0.67 ± 2.24	1.44 ± 2.13	3.15 + 2.48	−2.27 + 2.08
R^2^	19.60%	9.46%	15.37%	19.06%	4.49%
WAs∆R^2^	3.18%	2.48%	2.98%	1.97%	0.37%

*p < .05; **p < .01; ***p < .001; ****p < .0001 ; ^#^p < .10.

#### Socioeconomic associations

Maternal WASI scores and maternal education were inversely associated with WAs [*r*_(270)_ = −0.17, *p* < 0.01 and *r*_(270)_ *= −*0.18, *p* < 0.01, respectively], although not substantially so. WAs was not distributed randomly across the study area: the proportion of households with WAs ≥ 10 varied with school district [*χ*^2^_(2)_ = 4.89, *p* < 0.09]. There were also significant variations across districts (Table 3) in maternal IQ [*F*_(2,269)_ = 5.84, *p* < 0.005], and in the proportions of mothers with college degrees [*χ*^2^_(2)_ = 11.89, *p* < 0.005], proportions of HOME scores above the median (*χ*^2^_(2)_ = 30.99, *p* < .0001), and proportions of families reporting that their home included either a water filtration or treatment system [*χ*^2^_(2)_ = 8.68, *p* <0.02]. In each instance, more optimal conditions were seen among families in District A than in either other district. In contrast, although HOME scores were also associated with both maternal IQ and with School District, HOME score was not associated with WAs [*r*_(241)_ = −0.04].

**Table 3:** Sample characteristics by school district

**Characteristics**	**District “A” (n = 78)**	**District ”B” (n = 51)**	**District ”C” (n = 143)**
WAs ≥10 μg/L	21.8% (17)	37.3% (19)	34.3% (49)
HOME score			
≥53	61.5% (48)	51.0% (26)	40.6% (58)
<53	35.9% (28)	49.0% (25)	40.6% (58)
Missing 6+ items	2.6% (2)	0% (0)	18.9% (27)
Maternal education ≥ 16 years	64.1% (50)	33.3% (17)	51.1% (73)
Maternal IQ mean ± SD	118.47 ± 8.65	113.25 ± 11.95	113.43 ± 11.96
Home has filtration or water treatment system	68.0% (53)	52.9% (27)	47.6% (68)

Regardless of school district, mothers with college degrees or higher were significantly more likely to report that their well had been tested for WAs [68.6% vs. 50.8%: *χ*^2^_(2)_ = 9.02, *p* < 0.005] and that their home included either a water filtration or water treatment system [61.4% vs. 47.0%: *χ*^2^_(1)_ = 5.77, *p* < 0.02]. Maternal IQ was higher among those reporting well testing for WAs compared to those who had not tested [mean IQ = 116.3 vs. 112.7: *t*_(270)_ = 2.54, *p* < 0.02]. Compared to mothers who had completed college, those who had not were significantly more likely to report that the family relied on bottled water as an alternative source [34.3% vs. 51.5%: *χ*^2^_(1)_ = 8.28, *p* < 0.005]. Mothers who used bottled water had lower IQ scores than those who did not [mean IQ = 113.0 vs. 116.2: *t*_(270)_ = 2.29, *p* < 0.05]. We did not assess the quantity of bottled water usage.

### Covariate-adjusted associations between WAs and child intelligence (Model 2)

As Table 2 shows, both maternal intelligence and education (categorically defined) contributed significantly to most measures of child intelligence; associations were least strong for Processing Speed. Number of additional children in the home was not significantly associated with child intelligence. Children from families with missing data on the HOME obtained lower scores on some measures of child intelligence. School district was unrelated to child outcomes. Adjusting for the features in Model 2, results for WAs persisted, although effect sizes were expectably lower. WAs between 5 and 10 μg/L remained significantly associated with FSIQ and with all Index scores (except for Processing Speed). Just as in Model 1, significant associations for other levels of WAs concentration were relatively infrequent: WAs between 10 and 20 μg/L remained significantly associated with Perceptual Reasoning scores, and exposure to levels ≥ 20 μg/L remained negatively associated with Working Memory scores, although no longer statistically significant. On average, in adjusted analyses WAs explained 2-3% of the variance in child outcomes, except for Processing Speed, where its contribution was considerably lower. As in Models 1 and 2, there were no significant differences between estimated points lost across the three exposure groups, though power was limited, particularly in the two highest groups.

To examine differential associations at extremely low exposures, we reproduced these analyses, further dividing the < 5 μg/L group into two groups: WAs < 1 μg/L (n = 83) and ≤1 μg/L < WAs <5 μg/L (n = 58: data available upon request). There were no statistically significant differences in estimated IQ points lost for any subscale between these two groups. Results were unchanged when we removed school district from Model 2. Results were essentially unchanged when we repeated the analyses among children who lived at their present address for 5 or more years, although reduced power expectably lowered statistical significance (n = 215: data available upon request). Ln(WAs) was significantly associated with decrements in IQ in simple models only (data available upon request).

### Estimating IQ decrements attributable to WAs exposure

Based on Model 2, compared to those with WAs < 5 μg/L, children with WAs ≥ 5 μg/L showed reductions of 6.09 FSIQ points (95% CI = −9.99, −2.19; *p* < 0.01), 4.97 points in Perceptual Reasoning scores (95% CI = −9.18, −0.76; *p* < 0.05), 4.88 points in Working Memory scores (95% CI = −9.30, −0.46; *p* < 0.05) and 6.22 points in Verbal Comprehension scores (95% CI: −11.12, −1.32; *p* < 0.02). There were no statistically significant differences in estimated points lost among the exposure groups.

### Nail As and intellectual function

NAs and WAs were significantly correlated [Spearman *r*_(246)_ = 0.21, *p* < 0.001)]. NAs, however, was not significantly associated with changes in IQ scores, in either continuous or stratified models (data available on request).

## Discussion

In the US, approximately 13 million people are exposed to drinking water that exceeds the US standard [28]. Although the current EPA standard for As in drinking water is 10 μg/L [27], at least one state (New Jersey) has enacted a standard of 5 μg/L [29].

Adjusting for HOME scores, for maternal education and IQ, for school district, and for the number of other children in the home (Model 2), children exposed to WAs ≥ 5 μg/L from household wells (compared to WAs < 5 μg/L) showed significant reductions in Full Scale, Working Memory, Perceptual Reasoning and Verbal Comprehension scores. Categories of WAs exposure at levels higher than 5 μg/L did not differ significantly among themselves in their contributions to IQ score reductions. However, the small sample size may have hindered finding associations.

In Bangladesh, with similarly aged children, drinking from wells with more widely ranging WAs concentrations (0.1-790 μg/L), and using an earlier test version (WISC-III), we found negative associations between WAs and Performance scales that persisted upon adjustment for sociodemographic contributors [2], and observed similar results in Bangladeshi 6-year olds for Performance scores on the WPPSI-III [1]. More recently, we reported negative associations between As measured in blood and WISC-IV Working Memory; with adjustment for blood Mn, and for other contributors, this association remained marginally significant at *p* < 0.09 [3]. In re-standardization leading to the WISC-IV, certain WISC-III Performance subtests were re-organized into Working Memory, Processing Speed and Perceptual Reasoning domains (and other subtests revised, added or eliminated). The Digit Span subtest is common to both WISC-IV Working Memory and WISC-III Performance Scale. Collectively, our work in Bangladesh and in Maine suggests that aspects of Performance intelligence, particularly Perceptual Reasoning and Working Memory, are impacted by exposure to As in drinking water.

Among older adults [30], with adjustment for age, gender, education and ethnicity, WAs (mean WAs = 6.3 μg/L) was associated with a wide range of cognitive skills, including processing speed, executive function, and memory. Moreover, animal studies have shown a dose-dependent accumulation of As in many parts of the brain [31,32] that play important roles in human cognition and memory. In As-exposed rodents, morphological and neurochemical changes have been noted in the hippocampus, along with expectable learning and memory deficits [33,34].

### Comparisons with findings from Bangladesh

Cross-cultural accommodations in the measurement of intelligence in our earlier work prompted the present US replication. Our findings of adverse impact in a US sample, particularly in Performance-related functioning, gives confidence to the generalizability of findings from our work in Bangladesh, where we also observed a steep drop in intelligence scores in the very low range of WAs concentrations (see Figure one in our earlier work [2]).

Because there is no currently standardized test of child intelligence for use in Bangladesh, in earlier work we adapted widely-used instruments. Most particularly, we used weighted sums of items, rather than more commonly used IQ scores. Moreover, we dropped certain items from our battery because of differences in experience that were practically universal for our study children; as examples, rural Bangladeshi children are unfamiliar with Christopher Columbus (Information subscale). Most subtests were unchanged (e.g., Digit Span, Block Design); alterations affected three or fewer items or else the entire subtest was eliminated. While these changes were minimal, they added some measure of uncertainty.

Children in Bangladesh and in the US also differ in regularity of school attendance, health and nutritional status. Stunting has consequences for later intellectual function and school progress [35]. Almost 30% of children included in our Bangladesh studies would have been characterized as stunted [36]. The negative association between WAs and Performance intelligence in the Bangladesh sample persisted, even with adjustment for stunting [2].

Differences in the distributions of WAs between the present US sample and our earlier work with Bangladeshi children likely explain some points of contrast between present and earlier findings. In much of Asia, WAs levels range far higher than in the US. The differences across samples in exposure characteristics may have affected our ability to detect effects at all points across the exposure/outcome curve. We lacked high-end exposures in the Maine sample, and we had far fewer low-end exposures in Bangladesh. For example in our initial Bangladesh studies of 6- and 10-year old children, mean WAs levels were, respectively, 120.1 and 116.6 μg/L. To illustrate further, approximately 70% of the present sample were exposed to WAs < 10 μg/L, compared to 30% among our Bangladeshi 10-year-olds [2]. Differences are also substantial when we consider WAs < 5 μg/L (52% in Maine, 25% in Bangladesh), or WAs >20 μg/L (12% in Maine, 67% in Bangladesh). At the far lower levels reported here (mean WAs = 9.9 μg/L), we still detected an adverse association attributable to WAs for Index and FSIQ scores. Conceivably, a US sample with children exposed at the higher levels seen in Bangladesh might detect continued adverse associations at those levels.

### Magnitude of associations

With adjustment for other contributors, WAs ≥ 5 μg/L was associated with reductions of 4.5-6.5 points in FSIQ and in most Index scores. The magnitudes of these associations are similar to those observed with modest increases in blood lead, an established risk factor for diminished IQ. An increase in blood lead from 2.4 to 10 μg/dl or from 10 to 20 μg/dL is associated with estimated decrements of 3.9 and 1.9 IQ points, respectively [37]. Household use of the pesticide chlorpyrifos has been associated with a decline in FSIQ and Working Memory of 1.4 and 2.8 points, respectively, for each standard deviation of cord blood levels [38]. Among 7-year olds, prenatal (but not postnatal) exposure to organophosphate pesticides was associated with a loss of 7 FSIQ points, comparing highest to lowest quartile, as well as with all Index Scores [39].

Overall, the variance explained in predicting component indices of the WISC-IV was slightly smaller, relative to what we have noted in our work in Bangladesh (where R^2^ is generally in the range of 20%-30%), most likely a consequence of the lower and narrow range of exposures examined here.

### NAs as a biomarker of exposure

The significant correlation between NAs and home WAs levels suggests that household well WAs contributes to the body burden of As. On the other hand, NAs was not significantly associated with changes in IQ scores. This lack of a significant association might reflect that only 248 participating children provided nails, and that the range of NAs concentrations was narrow, so that we had less power to detect a small effect size. Alternatively, NAs may reflect exposure less accurately in children than in adults, due to the rapid growth of other organ systems in children that may alter the relative distribution of As across tissues.

### Limitations

Although we were able to characterize levels of As exposure in home drinking water, we did not collect information on quantity of water consumed. At the time of our study, public water sources, such as schools, were required to evaluate drinking water and to maintain WAs levels < 10 μg/L. Compared to circumstances in many other parts of the world, where children’s consumption can be directly linked to use of wells at home and/or at school, US children access water through a wide range of processed sources (bottled water, soft drinks), complicating consumption estimates. Scheduling home visits across our largely rural communities at times convenient for staff and families proved challenging, and resulted in children occasionally not being present during the home visit; defining these children as “Missing” on HOME allowed their retention in analyses, but we were unable to characterize the quality of the home environment for 29 families. Finally, in this cross-sectional study, we were unable to characterize WAs exposure retrospectively across the lifespan. We measured current well characteristics, and length of residence, but few children had resided in the present home for their entire lives. And while using “entire life residence” as a more stringent exclusion criterion would have better characterized exposure during period(s) of peak brain development, lack of residential mobility may be a marker for other social features not considered here. As we note, when we restricted analysis to the 215 children with 5 years of continuous residence, results were essentially unchanged.

### Do new analytic approaches need to be considered?

Since the 1980’s [40], the field of developmental neurotoxicology has relied upon descriptive models that adjust for other possible contributors to child intelligence, such as maternal intelligence and education. The underlying assumption in that approach is that these known predictors of child intelligence are not associated with exposure. In older studies, and in developing nations, this assumption likely remains reasonable. However, with growing public awareness of the hazards associated with certain environmental exposures, contemporary studies in developed nations need to be aware that maternal characteristics may influence exposure levels; for example, more educated mothers may be more likely to avoid exposure. Our observations have implications for the design of future studies of highly publicized potentially toxic exposures. We suggest that future studies collect data related to parental knowledge of exposure patterns and health risks of these agents. Such data can be used in deciding which parental factors to include in statistical models.

## Conclusions

Adjusting for HOME scores, maternal education and IQ, school district, and for the number of other children in the home, as compared to children with household well WAs < 5 μg/L, those exposed to WAs ≥ 5 μg/L showed significant reductions in Full Scale IQ scores and in most Indices, resulting in losses of 5–6 points. Categories of exposure at levels higher than 5 μg/L did not differ among themselves, nor did exposure categories at levels below 5 μg/L, suggesting that 5 μg/L may represent an important threshold. The strength of associations is similar to those observed with modest increases in blood lead, an established risk factor for diminished IQ.

## Abbreviations

As: Arsenic; EPA: Environmental Protection Agency; FSIQ: Full Scale IQ; HR ICP-MS: High-resolution inductively-coupled plasma mass spectrometry; IQ: Intelligence quotient; ME: Maine; Mn: Manganese; NAs: Nail arsenic; NH: New Hampshire; QC: Quality control; RSU: Regional School Unit; UAs: Urinary arsenic; WAs: Water arsenic; WPPSI-III: Wechsler Preschool and Primary Scale of Intelligence: Third Edition; WISC-III: Wechsler Intelligence Scale for Children-Third Edition; WISC-IV: Wechsler Intelligence Scale for Children-Fourth Edition.

## Competing interests

The authors declare they have no competing interests.

## Authors’ contributions

GW designed and trained on the cognitive assessment battery and helped draft the manuscript; XL conducted and interpreted the data analyses; NL conducted training on all aspects of the home visits and supervised field staff; JK and PF participated in the design and interpretation of the study and helped draft the manuscript; AV developed the methodology for water collection and analysis, supervised the laboratory analyses and participated in the interpretation of findings; JM conducted the water analyses; DL created and maintained the study database and participated in the statistical analyses; RA assisted in the design and recruitment procedures; AS supervised the data collection ; JG participated in the design and interpretation of the study and helped draft the manuscript; all authors read and approved the final manuscript.
